# Making useful clinical guidelines: the ESGAR perspective

**DOI:** 10.1007/s00330-019-6002-9

**Published:** 2019-02-07

**Authors:** A. A. O. Plumb, D. Lambregts, D. Bellini, J. Stoker, S. Taylor, ESGAR Research Committee, M. Ronot, A. Perez-Girbes, J. H. Runge, V. Vandecaveye, C. Zech

**Affiliations:** 1 Centre for Medical Imaging, University College London, London, UK; 2 Netherlands Cancer Institute, Amsterdam, The Netherlands; 3 Department of Radiological Sciences, Sapienza University of Rome, Rome, Italy; 4 Department of Radiology and Nuclear Medicine, Amsterdam UMC, Academic Medical Center, University of Amsterdam, Amsterdam, The Netherlands

Clinical guidelines are important and influential; they can improve processes involved in patient care, thereby also improving patient outcomes [1]. They are both commonly downloaded from journal websites and highly cited [2], helping clinical decision making and service commissioning. Yet, the quality of such guidelines is highly variable; a review of 279 guidelines published between 1985 and 1997 found that overall adherence to high-quality methodological standards was less than 50% [3]. Just as Altman [4] has argued that the misuse of statistics is unethical for primary research, it is equally inappropriate for guideline documents to recommend specific practices unless developed robustly and transparently. To do otherwise risks erroneous care, and, ultimately, patient harm. Readers of guidelines (clinicians, patients and policy-makers) require reassurance that these authoritative documents have identified, appraised and considered the available evidence, or draw attention to weaknesses in the literature if appropriate.

Accordingly, many organisations and professional societies issuing clinical guidelines have formalised their development process. This has several advantages, but perhaps most significantly, it permits methodological rigour to be embedded into the guideline development process in a consistent fashion. The organisation is able to stipulate, in advance, minimum standards for literature searching, evidence synthesis, construction and ratification of guideline statements; all of which would otherwise be at the whim of individual guideline development committees or groups. Yet, what makes a high-quality guideline? Fortunately, this question has been the subject of many years’ work by several organisations, notably the AGREE consortium, whose most recent iteration (AGREE II) encompasses 23 items that can be used to assess the quality of a particular clinical guideline [5]. As a by-product of this quality assessment tool, it provides a framework to permit high-quality guideline development and reporting [6].

The European Society of Gastrointestinal and Abdominal Radiology (ESGAR) has developed its own guideline development process, aiming to standardise and improve the quality and transparency of guidelines issued under its aegis. This was initiated in the mid-2017, and guidelines published from October 2018 onwards will adhere to its structure. The key components of this process, which adhere to the principles of AGREE II, are summarised in Fig. 1 and below; the full document and supporting information are available on the ESGAR website (https://www.esgar.org/guidelines-publications/research-committee/).

**Fig. 1:**
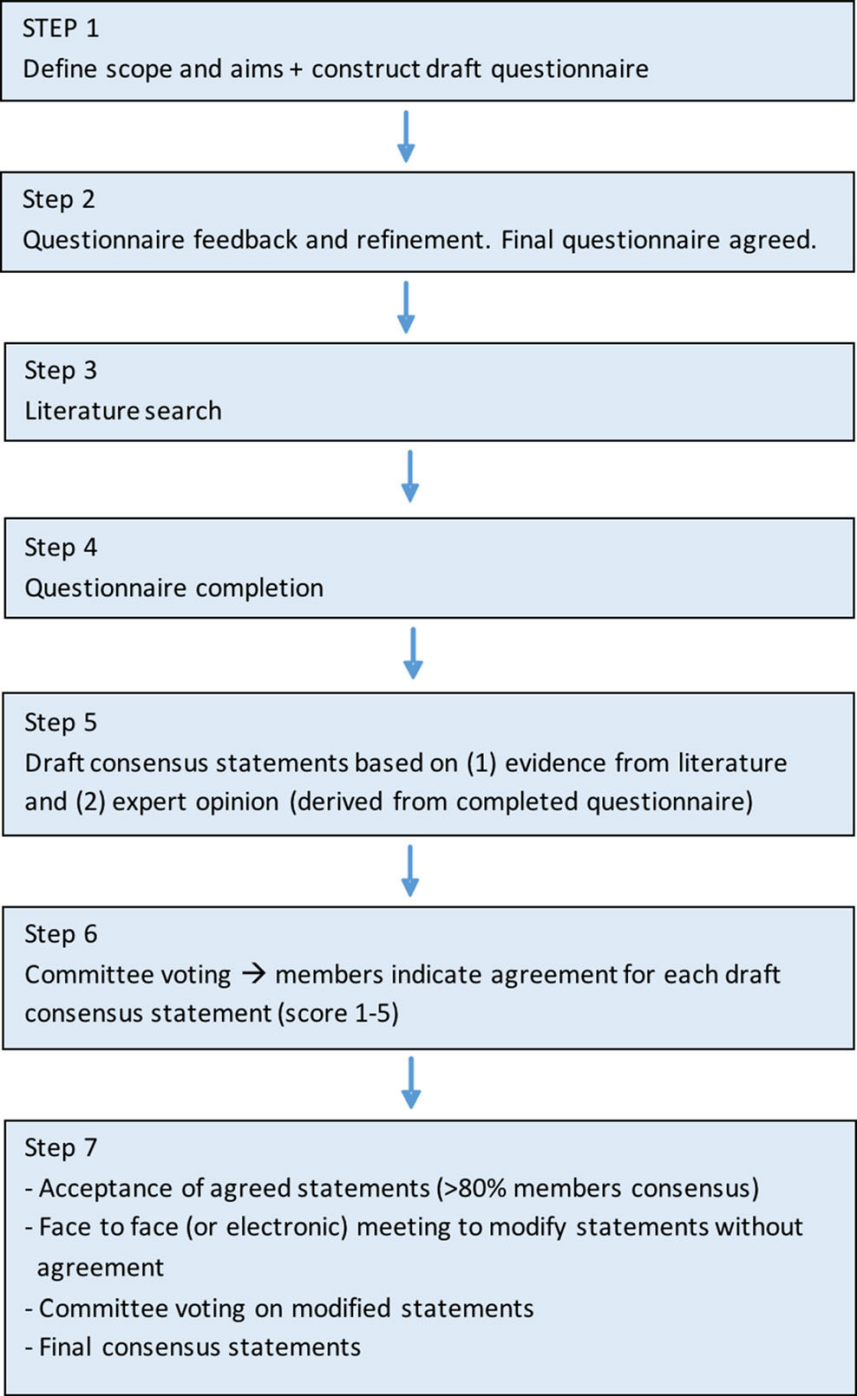
Flowchart of key steps in the ESGAR Guideline Development Process

## Guideline prioritisation

Potential topics for guidelines will be sought from the ESGAR membership, members and leaders of other societies and members of the public and journal editors. A form is available on the ESGAR website to assist this (at the same URL as listed above). Topics will be prioritised based on specific criteria, namely the burden of the relevant disease (or radiological technique), the impact that this has on radiological services, the extent of uncertainty in current clinical practice (including availability of other high-quality-related guidelines) and the availability of published evidence upon which a guideline could be based. The feasibility of developing a guideline using the resources and expertise of the ESGAR membership will also be considered. These criteria will be assessed by the ESGAR research committee before a new guideline is commissioned.

## Step by step consensus process

### Evidence selection and synthesis

The guideline development group will construct and circulate an initial questionnaire in order to identify the scope and aims of the guideline, and prioritise topics for detailed literature review. Thereafter, where possible these topics will be converted into specific clinical questions adopting the PICO format (patients/participants, intervention, control/comparators, outcomes). These clinical questions will then serve as the basis for the literature search. The literature search, including the databases used, search terms, inclusion dates and language restrictions, will be documented clearly and used uniformly by the guideline group. Relevant articles will be retrieved and summarised into evidence tables with accompanying explanatory text, including an assessment of the literature quality.

### Consensus statements and committee voting

Using the evidence synthesis tables and explanatory text, the guideline group will construct draft consensus statements for the final guideline. Where the evidence is contradictory or incomplete, relevant statements can still be constructed, but may need to be based on the group’s opinion with anonymous voting, using a modified Delphi process. Each voting member will be asked regarding their agreement with a given statement on a five-point scale (strongly disagree, disagree, neither agree nor disagree, agree and strongly agree). For a statement to achieve consensus, 80% of guideline group members must agree or strongly agree with that statement. Statements not achieving consensus initially may be modified and subjected to repeat voting; a maximum of three iterations is suggested as the upper limit before a statement is defined as failing to reach consensus. Statements that do not achieve consensus may be included in the Discussion of the final guideline document, but should not be recommended by the group.

## Guideline reporting, appraisal and updating

The final output of the process will be a guideline document, which will include its background, target audience and endorsing societies. The consensus statements themselves, with an associated level of evidence quality and strength of recommendation, will be provided, with the evidence summary tables and explanatory text made available as supplementary information. Discussion points should include statements that failed to reach consensus, recommendations for how the guideline should be implemented in clinical practice, where the literature review uncovered gaps and opportunities for further research, and a timeframe for updating the guideline. During the process of constructing the guideline document, the group will conduct an internal appraisal of methodological quality by using the AGREE II instrument and use this to guide their manuscript preparation. For example, target users of the guideline must be explicitly identified; statement wording should be clear and unambiguous; and the key recommendations must be easily identifiable. The key aspects of ESGAR guideline reporting are summarised in Table 1. Reports will be published in the peer-reviewed literature and made Open Access, to enhance visibility and likelihood of implementation.

**Table 1:** Key aspects of AGREE II to be incorporated into ESGAR guidelines publications and reports

Item	Original AGREE II item number
Domain 1: scope and purpose	
1. The overall objective(s) of the guideline must be specifically described.	1
2. The health question(s) covered by the guideline must be specifically described.	2
3. The population (e.g. patients with a particular condition or undergoing a certain test) to whom the guideline is meant to apply is specifically described.	3
Domain 2: stakeholder involvement^a^	
4. The guideline development group includes individuals from all the relevant professional groups and subspecialties.	4
5. The target users (e.g. radiologists and gastroenterologists) of the guideline will be clearly defined.	6
Domain 3: rigour of development	
6. Systematic methods must be used to search for evidence.	7
7. The criteria for selecting the evidence will be clearly described.	8
8. The strengths and limitations of the body of evidence will be clearly described.	9
9. The methods for formulating the recommendations must be clearly described.	10
10. The health benefits, side effects and risks will be considered in formulating the recommendations.	11
11. There is an explicit link between the recommendations and the supporting evidence.	12
12. All guidelines will be externally reviewed by experts prior to its publication, typically via peer-review and submission for publication.	13
13. A procedure for updating the guideline will be provided, either in the final guideline publication or via the ESGAR website.	14
Domain 4: clarity of presentation	
14. Recommendations must be specific and unambiguous.	15
15. The different options for management of the condition or health issue will be clearly presented.	16
16. Key recommendations should be easily identifiable and highlighted as such within the final published guideline	17
Domain 5: applicability	
17. The guideline should provide advice and/or tools on how the recommendations can be put into practice.	18
18. The guideline should describe facilitators and barriers to its application.	19
19. The potential resource implications of applying the recommendations should be considered.	20
20. The guideline should present monitoring and/or auditing criteria.	21
Domain 6: editorial independence	
21. The views of the funding body (i.e. the ESGAR executive) will not be permitted to influence guideline content.	22
22. Competing interests of guideline development group members will be recorded, addressed and published on the ESGAR website	23

^a^Where practicable, patient groups should be consulted during guideline construction (AGREE II item 5)

## Summary

Well-constructed clinical guidelines have the potential to substantially improve patient care across multiple institutions in different countries. To maximise their benefit, they must be prioritised, developed, disseminated and implemented using methodologically sound and transparent techniques. The ESGAR is committed to ensuring consistency and excellence in its guidelines, and all future ESGAR guidance will follow the principles outlined here.

## Electronic supplementary material

ESM 1 Full ESGAR guideline development process document (PDF 1031 kb)

ESM 2 Example search strategy for guideline evidence synthesis (PDF 445 kb)

ESM 3 Example evidence summary tables (PDF 1115 kb)

## References

[1] Lugtenberg M, Burgers JS, Westert GP (2009). Effects of evidence-based clinical practice guidelines on quality of care: a systematic review. Qual Saf Health Care.

[2] Baek S, Yoon DY, Lim KJ, Cho YK, Seo YL, Yun EJ (2018) The most downloaded and most cited articles in radiology journals: a comparative bibliometric analysis. Eur Radiol. 10.1007/s00330-018-5423-1 10.1007/s00330-018-5423-1 29736848

[3] Shaneyfelt TM, Mayo-Smith MF, Rothwangl J (1999). Are guidelines following guidelines? The methodological quality of clinical practice guidelines in the peer-reviewed medical literature. JAMA.

[4] Altman DG (1980). Statistics and ethics in medical research. Misuse of statistics is unethical. Br Med J.

[5] Brouwers MC, Kho ME, Browman GP (2010). AGREE II: advancing guideline development, reporting and evaluation in health care. CMAJ.

[6] Brouwers MC, Kerkvliet K, Spithoff K, Consortium ANS (2016). The AGREE Reporting Checklist: a tool to improve reporting of clinical practice guidelines. BMJ.

