# Supplementary material for: Making useful clinical guidelines: the ESGAR perspective
Source: Eur Radiol. 2019 Feb 7;29(7):3757–60. doi: 10.1007/s00330-019-6002-9 (PMC6554243; doi:10.1007/s00330-019-6002-9)
Supplement: Supplementary file 1 — Full ESGAR guideline development process document (PDF 1031 kb) [file 330_2019_6002_MOESM1_ESM.pdf]

ESGAR guidelines for development of consensus guidelines

Contents

Introduction (aims) 2

Guideline steering role of the Research Committee 2

Guideline formats 3

A. Monodisciplinary: 3

B. Multidisciplinary: 3

Selection of new guideline topics 4

Development process for monodisciplinary guidelines 5

A. Consensus guideline group selection 5

B. Step-by-step consensus process 7

Step 1 – Define scope and aims & construct draft questionnaire 8

Step 2 – Refine & construct final questionnaire 9

Step 3 – Literature search 9

Step 4 – Questionnaire completion 10

Step 5 – Draft consensus statements 10

Step 6 – Committee voting 14

Step 7 – Face to face meeting & construction of final consensus statements 14

C. Critical appraisal of guideline (AGREE II reporting checklist) 15

Manuscript preparation, publication and dissemination 15

References 17

Introduction (aims)

This document details the methodology for the development of ESGAR guidelines, as proposed by the ESGAR Research committee. The aims are to ensure consistency and excellence, by providing a detailed framework for the creation and dissemination across the range of ESGAR guidelines, such that they are viewed as being among the methodologically highest quality guideline(s) available.

Guideline steering role of the Research Committee

One of the roles of the ESGAR Research Committee is to initiate, coordinate and oversee the production and progress of ESGAR guidelines through to their completion and publication. Hitherto, this role has been conducted by the ESGAR Guidelines Steering Group, the function of which has been incorporated into the Research Committee since 2017. The Research Committee comprises a chair and committee members who are appointed to the Research Committee at the recommendation of the Executive Committee. The list of current members is maintained on the ESGAR website.

Regarding ESGAR guidelines, the tasks of the Research Committee are:

- To identify potential topics for new ESGAR guidelines internally.
- To receive and prioritise topics for new ESGAR guidelines from other external sources, including the Executive Committee, the ESGAR membership, other societies, and members of the public.
- To co-ordinate and promote collaboration with other radiological and medical societies for guideline development.
- To assist with methodological and scientific development of ESGAR guideline documents.
- To nominate a suitable ESGAR member to chair a planned guideline on behalf of the ESGAR.
- To participate in guideline publication, wider dissemination and support initiatives for their clinical implementation.
- To organise the review and updating of existing guidelines if needed.

The Research Committee Chair will report to the ESGAR Executive Committee.

Guideline formats

The ESGAR Research Committee envisions two main types of guideline:

A. Monodisciplinary:

- Monodisciplinary guidelines are developed under sole ESGAR stewardship (or in collaboration with fellow radiological societies), and will typically concern technical performance and protocols relevant to abdominal imaging using high technology imaging platforms and/or their clinical deployment.
- Monodisciplinary guidelines can generally be developed relatively rapidly (as input from non-radiological societies is usually not required), which is desirable so that the relevant guideline is not overtaken by parallel technological or clinical advances.
- The guideline development process for monodisciplinary guidelines is described in detail below (see section 'Guideline development process').
- Examples of completed and published monodisciplinary guidelines under sole ESGAR stewardship include the ESGAR CT colonography consensus documents, the ESGAR consensus documents on MRI in rectal cancer and the ESGAR consensus statements on liver MR imaging and clinical use of liver-specific contrast agents (1-3). Examples of guidelines published by ESGAR in collaboration with other radiological societies are the ESGAR/ESPR consensus statement on cross sectional bowel imaging and the ESUR/ESGAR MRI of pelvic floor dysfunction guidelines (4, 5)

B. Multidisciplinary:

- Multidisciplinary guidelines will normally be developed in collaboration with one or more relevant partner clinical societies. They typically describe the role of abdominal imaging in specific clinical circumstances and diseases, including comparison of multiple imaging techniques where appropriate.
- Generally, the clinical society will be lead guideline development, and will therefore adopt its own favoured methodology. Normally, ESGAR members co-opted to help will follow the lead society's guideline development strategy, provided that doing so will lead to a high-quality guideline. The selection process for such individuals to represent ESGAR as delegates

is detailed further below. The final decision regarding which ESGAR member will be put forward to represent the society will rest with the Research Committee.

- ESGAR delegates for multidisciplinary guidelines should ensure that clear authorship arrangements are made before participating in the guideline process. There should be full and appropriate acknowledgement of ESGAR's involvement in the published guideline.
- It is possible that ESGAR will be the lead organisation in collaborations with other societies, in which case the guideline will follow the development process detailed in this document (see 'Guideline development process' section).
- Examples of multidisciplinary guidelines include the ESGE/ESGAR guidelines for CT colonography, the ESGAR/EAES/EFISDS/ESGE guidelines on gallbladder polyps, and the ECCO/ESGAR guidelines for imaging of inflammatory bowel disease (4-8).

Selection of new guideline topics

Potential topics for new ESGAR monodisciplinary guidelines will be identified via several routes: as a recommendation from the Executive Committee or Research Committee; as a result of approach from other medical or radiological societies; following feedback from the ESGAR annual meeting or from the editorial boards of the journals *European Radiology* and *Insights Into Imaging*; or directly from members of the society. To facilitate the latter, a form is available on the ESGAR website to permit members to propose topics to the Chair of the Research Committee. New proposals for guidelines will be discussed by the Research Committee and prioritised based on the following criteria: (a) burden of the relevant disease, (b) impact and burden on gastrointestinal and abdominal radiological services, (c) extent of uncertainty in current clinical practice, (d) availability of existing guidance, (e) availability of evidence on which to produce a meaningful guideline and (f) capacity and expertise available within the ESGAR membership. The Research Committee may choose to undertake a provisional scoping review of the literature before a final decision is made regarding whether or not to proceed with a proposed guideline.

Development process for monodisciplinary guidelines

Monodisciplinary guidelines (or multidisciplinary guidelines led by ESGAR) typically include one or both of the following two components: technical guidelines (i.e. how to perform a certain imaging technique or procedure) and clinical guidelines (i.e. how to deploy a certain technique(s) in clinical practice). Monodisciplinary guidelines and multidisciplinary guidelines led by ESGAR should adhere to the guideline development steps described in detail below, consisting of the following three parts: (A) Guideline group selection, (B) Step-by-step consensus process and (C) Critical appraisal of the guideline using the AGREE II reporting checklist.

A. Guideline group selection

For each guideline, a guideline group will be assembled, consisting of one (or two) chairs, an expert panel (i.e. these will constitute the voting group members) and optionally one or more research fellows.

- The chair of the guideline group for each guideline will be selected by the ESGAR Research Committee, based on (a) publication record in the field, (b) clinical expertise regarding the guideline topic, (c) geographical location, to ensure (as far as possible) appropriate representation across the ESGAR membership and a broad range of expertise within the overall group, and (d) potential conflicts of interest (which may include participation in similar guidelines led by other organisations).
- A call for expressions of interest to take part in the guideline group may be circulated to all ESGAR members. From those expressing interest, a suitable number of expert panel members (who will take part in the consensus voting) will be selected by the guideline group chair using the same criteria as above.
- Consideration will be made to inviting representatives from sister organisations (for example paediatric or molecular imaging) if relevant to the guideline topic and content,

although in general it is expected this will be the exception rather than the rule for monodisciplinary guidelines.

- Patient representative(s) might be included as part of the guideline working group or be asked as external advisors when considered appropriate.
- Research fellows (if available) may be appointed to the guideline group to help with some of the tasks, for example the literature research and draft document construction. However, research fellows will generally not take part in the group consensus voting. Exceptions will be at the discretion of the chair of the relevant guideline group.
- For multi-disciplinary guidelines where ESGAR is the lead organisation, selection of group members from the other societies will in general adhere to the processes of these individual societies. The chair of the ESGAR component of the group will however liaise with the other society leads to ensure a balanced representation in the final committee structure.
- For multi-disciplinary guidelines where ESGAR is not the lead organisation, the Research Committee will nominate individuals to represent ESGAR as delegates; this process is a requirement for a guideline to be described and publicised as a joint ESGAR guideline. Selection of ESGAR delegates for multidisciplinary guidelines will be based on the same criteria as for ESGAR-led monodisciplinary guidelines, and will be open to all ESGAR members. In exceptional circumstances, for example where urgent delegate selection is needed, the Research Committee will nominate a representative directly.

The chair may choose to allocate guideline group members into smaller working groups (WGs) to lead specific topics within the guidelines. Each WG (or the chair) should nominate a lead, who will be responsible for coordinating the work of that WG and submitting the final output to the guideline group chair and the remaining group members. All group members should familiarise themselves with the AGREE II (Appraisal of Guidelines, Research and Evaluation) reporting checklist, a widely

used standard for assessing the methodological quality of practice guidelines (9, 10) before starting the consensus process (see section C below).

B. Step-by-step consensus process

A modified Delphi approach based on the RAND-UCLA appropriateness should be utilised, encompassing a detailed literature review and collective judgement of experts, including electronic and face to face discussion (11). A summary of the process is given in Figure 1 and individual steps are addressed in detail below. The guideline group or its chair may choose to defer from one or more steps in the consensus process provided that a clear rationale supporting this decision is presented to and explicitly approved in writing by the Research Committee.

Figure 1. Summary of consensus process

STEP 1
Define scope and aims + construct draft questionnaire

↓

Step 2
Questionnaire feedback and refinement. Final questionnaire agreed.

↓

Step 3
Literature search

↓

Step 4
Questionnaire completion

↓

Step 5
Draft consensus statements based on (1) evidence from literature and (2) expert opinion (derived from completed questionnaire)

↓

Step 6
Committee voting → members indicate agreement for each draft consensus statement (score 1-5)

↓

Step 7
- Acceptance of agreed statements (>80% members consensus)
- Face to face (or electronic) meeting to modify statements without agreement
- Committee voting on modified statements
- Final consensus statements

Step 1 – Define scope and aims & construct draft questionnaire

The guideline group will meet (face to face or electronically if a face to face meeting is not possible) to define the scope and aims of the guideline document. Under the direction of the chair, the group (subdivided into WGs if applicable) will produce an initial detailed questionnaire containing all items for which a consensus statement is planned. Each item on the questionnaire will consist of a specific

question with an appropriate range of possible responses, including an option for free text/comments. Items in the questionnaire should be grouped according to sub-topics (allocated to the individual WGs), for example patient preparation or MRI sequences.

Step 2 – Refine & construct final questionnaire

The draft questionnaire is distributed amongst all guideline group members allowing them to comment on the items included to ensure they fully align with the purpose and scope of the guideline. The questionnaire should then be modified based on feedback from all guideline group members and then sent to the Research Committee for information.

Step 3 – Literature search

A detailed literature search is performed in order to establish the evidence base pertaining to the individual items included in the final questionnaire. Where practicable, this will be achieved by converting individual questionnaire items into clinical questions to be answered by literature review. These questions should – if possible – be framed using the PICO (Patients / Participants, Intervention, Control / Comparators, Outcomes) format. The literature search should be performed by individuals appointed by the guideline group (for example research fellows) or by the group members themselves (split into WGs if applicable). The search strategy, including chosen databases, search terms, inclusion dates and language restrictions, must be clearly documented and used uniformly by the appointed committee members and/or WGs. The final search results, ideally presented as evidence tables summarising key references, should be circulated to all guideline group members, along with full abstracts/papers as appropriate. Example templates of both search strategies and evidence tables will be made available on the ESGAR website. Guideline group members and/or WGs are at liberty to further update the literature search at their discretion,

particularly in the face of new or emerging evidence, following approval by the guideline group Chair.

Step 4 – Questionnaire completion

The final questionnaire as agreed on in Step 2 is circulated to and completed by all voting committee members. Completed questionnaires are then collected by the committee Chair and a summary of the committee members' responses should be drafted by designated committee members or the ESGAR office. This summary document should then be circulated to all committee members.

Step 5 – Draft consensus statements

The questions in the final approved questionnaire are drafted into individual consensus statements with supporting text by the lead members of the working groups (if applicable) or by designated committee members. The process is informed by the outcomes of the detailed literature review (see Step 3) and/or by answers to the final questionnaire submitted by all members of the committee (see Step 4), according to the following guidelines:

- Consensus statements should primarily be based on the outcomes of the literature review even if this contradicts the results from the questionnaire, providing the literature is deemed to be of sufficient quality to guide best practice. Key references supporting statements should be graded for quality using levels of evidence as provided by the Oxford Centre for Evidence Based Medicine (www.cebm.net; see Table 1)

- When the available literature is deemed to be limited and/or of low quality, the committee may base statements on consensus opinion (derived from the completed questionnaires) even if these contradict the available low quality evidence, and justify this in explanatory text.

- If, for some items included in the circulated questionnaire, there is no available adequate literature to guide consensus statements, statements should be based on the opinion of the whole committee by selecting the favoured response (preferably by at least 50% of the committee members) provided to the questionnaire. Should there be no clear favoured response amongst the committee, a range of options or a more general overview statement may be provided. Detail/examples of good practice to support this more general statement should be provided in the explanatory text.
- Finally, a strength of recommendation should be provided for each statement using a binary classification (strong of weak), as described in Table 2 derived from '*Atkins et al. Grading quality of evidence and strength of recommendations*'(12).
    - N.B. In some cases the Oxford level of evidence may be relatively weak for a particular technique / intervention but nonetheless merit a strong recommendation. An example of such as case (from the Clinical indications for computed tomographic colonography: European Society of Gastrointestinal Endoscopy (ESGE) and European Society of Gastrointestinal and Abdominal Radiology (ESGAR) Guideline) is the recommendation not to perform CT colonography in patients with active colitis. Although this statement is not supported by high level evidence, it still merited a strong recommendation.

Table 1 – Oxford centre for Evidence-based Medicine Levels of evidence

	Therapeutic studies — investigating the results of treatment	Prognostic studies — investigating the effect of a patient characteristic on the outcome of disease	Diagnostic studies — investigating a diagnostic test	Economic and decision analyses — developing an economic or decision model
**Level I**	• High-quality randomised controlled trial with statistically significant difference or no statistically significant difference but narrow confidence intervals<br>• Systematic review (*a*) of level-I randomised controlled trials (and study results were homogeneous (*b*))	• High-quality prospective study (*c*) (all patients were enrolled at the same point in their disease with ≥80% follow-up of enrolled patients)<br>• Systematic review (*a*) of level-I studies	• Testing of previously developed diagnostic criteria in series of consecutive patients (with universally applied reference gold standard)<br>• Systematic review (*a*) of level-I studies	• Sensible costs and alternatives; values obtained from many studies; multiway sensitivity analyses<br>• Systematic review (*a*) of level-I studies
**Level II**	• Lesser-quality randomised controlled trial (eg, <80% follow-up, no blinding, or imperfect randomisation)<br>• Prospective (c) comparative study (d)<br>• Systematic review (*a*) of level-II studies or level-I studies with inconsistent results	• Retrospective study(*e*)<br>• Untreated controls from a randomised controlled trial<br>• Lesser-quality prospective study (eg, patients enrolled at different points in their disease or <80% follow-up)<br>• Systematic review (*a*) of level-II studies	• Development of diagnostic criteria on basis of consecutive patients (with universally applied reference gold standard)<br>• Systematic review (a) of level-II studies	• Sensible costs and alternatives; values obtained from limited studies; multiway sensitivity analyses<br>• Systematic review (*a*) of level-II studies
**Level III**	• Case-control study (*f*)<br>• Retrospective (*e*) comparative study(*d*)<br>• Systematic review (*a*) of level-III studies	• Case-control study (*f*)	• Study of non-consecutive patients (without consistently applied reference gold standard)<br>• Systematic review (*a*) of level-III studies	• Analyses based on limited alternatives and costs; imperfect estimates<br>• Systematic review (*a*) of level-III studies
**Level IV**	• Case series (*g*)	• Case series	• Case-control study<br>• Poor reference standard	• No sensitivity analyses
**Level V**	• Expert opinion	• Expert opinion	• Expert opinion	• Expert opinion

a. A combination of results from two or more prior studies.
b. Studies provided consistent results.
c. Study was started before the first patient enrolled.
d. Patients treated one way compared with patients treated another way at the same institution.
e. Study was started after the first patient enrolled.
f. Patients identified for the study on the basis of their outcome, called cases, are compared with those who did not have the outcome, called controls.

Table 2 – Strength of recommendation (adapted from Atkins et al. BMJ 2004)

Strength of recommendation	
Strong	Benefits clearly outweigh risks and burden, or vice versa. Usually stated as "we recommend"
Weak	Benefits closely balance with risks and burden, or vice versa. Usually stated as "we suggest"

Based on the above, the responsible committee members and/or WGs will create a document for incorporation into a first draft of the consensus statements which will be circulated to all committee members.

This output document should:

- List all individual consensus statements including strength of recommendation.
- Provide a table limited to key references detailing the best evidence available supporting each individual statement including the journal reference, a very brief description of findings, with Oxford level of evidence. An example of such a Table is presented in Table 3.
- Provide a short text summary of the evidence supporting each statement, or explaining the lack of evidence where there is none available.

Table 3 – example of key reference and evidence presentation

Reference	Brief description	Oxford evidence level
Cronin CG et al. MRI small-bowel follow-through: prone versus supine patient positioning for best small-bowel distention and lesion detection. AJR 2008; 191(2):502-6	40 patients underwent supine and prone MRI. Prone position had significantly higher distention scores but this did not translate into improved lesion detection or characterization	III
Gourtsoyiannis N, et al. MR enteroclysis protocol optimization: comparison between 3D FLASH with fat saturation after intravenous gadolinium injection and true	21 patients underwent MReCly. Image quality of True FISP compared with 3D FLASH. The true FISP sequence provided images with significantly fewer motion artifacts, whereas 3D FLASH was less sensitive to susceptibility and chemical shift artifacts	III

FISP sequences. Eur Radiol. 2001;11(6):908-13		
Froehlich JM, et al. Peristaltic effect of hyoscine N-butylbromide versus glucagon on the small bowel assessed by MRI. Eur Radiol. 2009 Jun;19(6):1387-93	10 volunteers underwent MRE after 40mg buscopan or 1mg glucagon. Aperistalsis lasted a mean of 6.8 min after buscopan compared with 18.3 after glucagon ($p < 0.0001$). In 50% of cases HBB did not accomplish aperistalsis, whereas glucagon always succeeded ($p = 0.05$).	IV

Step 6 – Committee voting

All guideline group members should grade their agreement with each draft consensus statement from 1 to 5 according to the following definitions; 1-strongly disagree, 2-somewhat disagree, 3-undecided, 4-somewhat agree, 5-strongly agree. Group members return their scores to the guideline group chair (or another responsible member). Responses will be summarised by designated group members tasked with this job, or by the ESGAR office.

Step 7 – Face to face meeting & construction of final consensus statements

Those statements achieving a score of 4 or 5 by at least 80% of guideline group members in Step 6 should be accepted into the final set of consensus statements. Those not achieving consensus should be re-discussed, ideally at a face to face meeting of the guideline group, or electronically if this is not possible. Statements not achieving consensus should be reviewed with reference to the literature summaries produced in Step 3, questionnaire responses and group member opinion. The statement is then either modified or deleted if it is clear consensus cannot be reached. Additional statements not covered by the original questionnaire content are permitted at this stage if deemed of sufficient importance following panel discussion. The list of revised and/or added statements should then be recirculated to the whole group to score agreement from 1 to 5 as in Step 6. Those statements achieving a score of 4 or 5 by at least 80% of guideline group members should be added to the final set of consensus statements. In general, a maximum number of 2 to 3 iterations should be allowed to reach a final consensus. If consensus is not reached within these rounds, the

statement is classified as not having reached consensus. Statements not achieving the a priori level of agreement defined above may be included as Discussion Points, since the 80% threshold is fundamentally an arbitrary cut-off.

C. Critical appraisal of guideline (AGREE II reporting checklist)

The guideline group should conduct a critical appraisal of their guideline using the AGREE II (Appraisal of Guidelines, Research and Evaluation) reporting checklist, a widely used standard for assessing the methodological quality of practice guidelines (9, 10). The AGREE II reporting checklist is intended to improve the comprehensiveness, completeness, and transparency of reporting in practice guidelines and will guide manuscript preparation. The AGREE II checklist consists of 23 items (structured according to six quality domains) and is freely available as a fill-able PDF or Microsoft word download via https://www.agreetrust.org/resource-centre/agree-reporting-checklist/.

A completed copy of the AGREE II checklist should be provided to the ESGAR Research Committee and in case of any major deviations from the AGREE II guidance, a clear rationale supporting this decision should be presented to and approved by the Chair of the Research Committee before proceeding to publication.

Manuscript preparation, publication and dissemination

In general, the guideline group will nominate individual(s) who will produce the final consensus guideline document to be reviewed and approved by the other group members.

The title of the consensus document should specify the topic and the names of the societies involved in guideline development and publication. All the members of the guideline group will be listed as authors.

The main text should contain, as a minimum, the following sections:

- An introduction presenting the background to the guideline, its target audience and endorsing societies;
- A methods section (which may refer to this document and key deviations from it);
- Any Working Groups formed within the main consensus committee, their composition and remit;
- Consensus statements, with their associated evidence level and strength of recommendation;
- A discussion (either as a separate section of the document or accompanying each consensus statement). The discussion section should ideally address:
    - the key findings of the consensus statements;
    - recommendations for how ESGAR guidelines can be implemented in clinical practice;
    - recommendations for future research (i.e. where are the literature gaps that the evidence review has uncovered)
    - recommendations for guideline review and updating (see also AGREE II checklist)
- A declaration of interests statement;
- References;
- Tables;
- Links to online appendices, which should include
    - A table listing each consensus guideline group member, potential Conflicts of Interest (COI) and (if relevant) the Working Group to which they were assigned;
    - The details of the literature review strategy, in sufficient detail to permit replication;
    - Summary output of the literature search including evidence tables for each questionnaire item considered by the group;

- A full list of items considered by the guideline group including the level of agreement reached (described as a simple percentage) for each item;
- A copy of the AGREE II reporting checklist (for monodisciplinary guidelines).

An ESGAR guideline should be published as a peer-reviewed indexed journal paper in all circumstances, to enhance international visibility and access. *European Radiology* should be considered, with *Insights into Imaging* or *Abdominal Radiology* as potential alternatives. All guidelines should be made publicly available through 'Open Access'. If required, open access fees will be covered by the ESGAR office (this should be arranged in advance). In addition, all ESGAR guideline documents will be made freely available from the ESGAR website (https://www.esgar.org/guidelines-publications/published-consensus-statements-guidelines/).

References

1. Neri E, Halligan S, Hellstrom M, Lefere P, Mang T, Regge D, et al. The second ESGAR consensus statement on CT colonography. Eur Radiol. 2013;23(3):720-9.
2. Beets-Tan RGH, Lambregts DMJ, Maas M, Bipat S, Barbaro B, Curvo-Semedo L, et al. Magnetic resonance imaging for clinical management of rectal cancer: Updated recommendations from the 2016 European Society of Gastrointestinal and Abdominal Radiology (ESGAR) consensus meeting. Eur Radiol. 2017.
3. Neri E, Bali MA, Ba-Ssalamah A, Boraschi P, Brancatelli G, Alves FC, et al. ESGAR consensus statement on liver MR imaging and clinical use of liver-specific contrast agents. Eur Radiol. 2016;26(4):921-31.
4. Taylor SA, Avni F, Cronin CG, Hoeffel C, Kim SH, Laghi A, et al. The first joint ESGAR/ ESPR consensus statement on the technical performance of cross-sectional small bowel and colonic imaging. European radiology. 2017;27(6):2570-82.
5. El Sayed RF, Alt CD, Maccioni F, Meissnitzer M, Masselli G, Manganaro L, et al. Magnetic resonance imaging of pelvic floor dysfunction - joint recommendations of the ESUR and ESGAR Pelvic Floor Working Group. Eur Radiol. 2017;27(5):2067-85.
6. Panes J, Bouhnik Y, Reinisch W, Stoker J, Taylor SA, Baumgart DC, et al. Imaging techniques for assessment of inflammatory bowel disease: joint ECCO and ESGAR evidence-based consensus guidelines. Journal of Crohn's & colitis. 2013;7(7):556-85.
7. Spada C, Stoker J, Alarcon O, Barbaro F, Bellini D, Bretthauer M, et al. Clinical indications for computed tomographic colonography: European Society of Gastrointestinal Endoscopy (ESGE) and European Society of Gastrointestinal and Abdominal Radiology (ESGAR) Guideline. Eur Radiol. 2015;25(2):331-45.
8. Wiles R, Thoeni RF, Barbu ST, Vashist YK, Rafaelsen SR, Dewhurst C, et al. Management and follow-up of gallbladder polyps : Joint guidelines between the European Society of Gastrointestinal and Abdominal Radiology (ESGAR), European Association for Endoscopic Surgery and other

Interventional Techniques (EAES), International Society of Digestive Surgery - European Federation (EFISDS) and European Society of Gastrointestinal Endoscopy (ESGE). Eur Radiol. 2017;27(9):3856-66.
9. Brouwers MC, Kerkvliet K, Spithoff K, Consortium ANS. The AGREE Reporting Checklist: a tool to improve reporting of clinical practice guidelines. BMJ. 2016;352:i1152.
10. Brouwers MC, Kho ME, Browman GP, Burgers JS, Cluzeau F, Feder G, et al. AGREE II: advancing guideline development, reporting and evaluation in health care. CMAJ. 2010;182(18):E839-42.
11. Fitch K, Bernstein S, Aguilar M, Burnhand B, LaCalle J, Lazaro P, et al. The RAND/UCLA Appropriateness Method User's Manual. AHCPR Pub No 95-0009 Rockville, MD: Public Health Service, US Department of Health and Human Services. 2001.
12. Atkins D, Best D, Briss PA, Eccles M, Falck-Ytter Y, Flottorp S, et al. Grading quality of evidence and strength of recommendations. BMJ. 2004;328(7454):1490.
