# Supplementary material for: Making useful clinical guidelines: the ESGAR perspective
Source: Eur Radiol. 2019 Feb 7;29(7):3757–60. doi: 10.1007/s00330-019-6002-9 (PMC6554243; doi:10.1007/s00330-019-6002-9)
Supplement: Supplementary file 2 — Example search strategy for guideline evidence synthesis (PDF 445 kb) [file 330_2019_6002_MOESM2_ESM.pdf]

Example search strategies:

Guideline: The first joint ESGAR/ ESPR consensus statement
on the technical performance of cross-sectional small bowel and colonic imaging

Guideline type: Monodiscplinary, technical

Reference: Taylor et al Eur Radiol (2017) 27:2570-2582

Search strategy used:

Search details	Time period: January 1983–December 2015
**Medline search**	
1	Crohn’s disease
2	Crohn [tiab]
3	Inflammatory bowel disease
4	1 OR 2 OR 3
5	Computed tomography
6	CT [tiab]
7	MRI
8	“Magnetic resonance” [All fields] OR (“magnetic” [All fields] AND “resonance” [All fields])
9	Ultrasound
10	5 OR 6 OR 7 OR 8 OR 9
11	4 AND 10
**Embase search**	
1	Crohn’s disease.ab,ti,sh,kw
2	Inflammatory bowel disease.ab,ti,sh,kw
3	1 OR 2
4	Computer Assisted Tomography.ab,ti,sh,kw
5	Exp Computer Assisted Tomography/

Search details	Time period: January 1983–December 2015
6	Nuclear magnetic resonance imaging.ab,ti,sh,kw
7	Exp Nuclear magnetic resonance imaging/
8	Echography.ab,ti,sh,kw
9	Exp echography/
10	4 OR 5 OR 6 OR 7 OR 8 OR 9
11	3 AND 10
**Cochrane search**	
1	Crohn disease [Mesh]
2	Inflammatory bowel disease [Mesh]
3	1 OR 2
4	Diagnostic techniques and procedures [Mesh]
5	3 AND 4

Guideline: Management and follow-up of gallbladder polyps

Guideline type: Multidisciplinary, ESGAR-led

Reference: Wiles et al Eur Radiol (2017) 27:3856-3866

Search strategy used:

MEDLINE and EMBASE databases were searched using search terms “gallbladder” and “polyp”, excluding case studies and limited to English language papers. Abstracts of all articles published between January 1995 and October 2015 were then reviewed.

1	Medline	(“gall bladder*” OR gallbladder*).ti	17699
2	Medline	polyp*.ti	60841
3	Medline	1 AND 2	363
4	Medline	3 NOT “case report*”	346
5	Medline	4 [Limit to: (Language English)]	250
6	EMBASE	4 [Limit to: (Language English)]	248

7	Medline, EMBASE	Duplicate filtered: [4 [Limit to: (Language English)]], [4 [Limit to: (Language English)]]	498<br><br>493 Unique results<br><br>5 Duplicate results
