# Supplementary material for: Making useful clinical guidelines: the ESGAR perspective
Source: Eur Radiol. 2019 Feb 7;29(7):3757–60. doi: 10.1007/s00330-019-6002-9 (PMC6554243; doi:10.1007/s00330-019-6002-9)
Supplement: Supplementary file 3 — Example evidence summary tables (PDF 1115 kb) [file 330_2019_6002_MOESM3_ESM.pdf]

Example Evidence Tables for clinical questions in ESGAR guidelines

Guideline: Clinical indications for computed tomographic colonography: European Society of Gastrointestinal Endoscopy (ESGE) and European Society of Gastrointestinal and Abdominal Radiology (ESGAR) Guideline

Guideline type: Multidisciplinary, ESGAR and ESGE joint leads

Reference: Spada et al Eur Radiol (2015) 25:331-345

Evidence table for clinical question 2 (CTC after incomplete colonoscopy):

Appendix e2 Computed tomographic colonoscopy (CTC): evidence tables

Table e1 Grading of studies comparing diagnostic yield of computed tomographic colonoscopy (CTC) after an incomplete colonoscopy.

Reference<br>First author	GRADE characteristics					Participants, n	Examinations after incomplete colonoscopy, n	Single- or multicenter	Catharsis	Tagging	Reconstruction interval, mm	Intravenous contrast	Type of CT	Dose, mAs<br>Supine	Reader experience	Reading strategy	CAD	Polyps ≥10 mm		Polyps ≥6mm		Advanced neoplasias/cancers		Extracolonic findings (specify use E-RADS)	Comments
Year	Study design	Study quality	Consistency	Directness	Other modifying factors									Prone				n	Per-patient PPV	n	Per-patient PPV	n	Per-patient PPV		
Pullens 36]<br>2013	Cohort<br>Retrospective	No serious limitations	Good	Good	None	136	136	Single	Yes (41.2%)	Yes (58.8%)	1	Yes	16- or<br>64-slice	100 mAs<br>200 mAs	Expert	n.a.	No	n.a.	n.a.	13<br>(additional yield)	n.a.	4 CRC (2.9%)<br>(additional yield)	n.a.	8 (5.9%)	None
Salamone [37]<br>2011	Cohort<br>Retrospective	No serious limitations	Good	Good	None	68	68	Single	Yes	No	1	No	16-slice	30 mAs<br>50 mAs	n.a.	n.a.	n.a.	8	n.a.	12	n.a.	0	n.a.	44 (64.7%)	None
Neerincx [38]<br>2010	Cohort<br>Retrospective	Only 14 patients underwent CTC	Good	Good	None	511	278	Multi	n.a.	n.a.	n.a.	n.a.	n.a.	n.a.	n.a.	n.a.	n.a.	n.a.	n.a.	n.a.	n.a.	1 CRC	n.a.	n.a.	Data reported on 14 patients
Iafrate 39]<br>2008	Cohort<br>Prospective	No serious limitations	Good	Good	None	136	136	Single	Yes	Yes	1	Yes<br>-20.50%	64-slice	50 mAs<br>100 mAs	Expert	Primary 2D	No	9	n.a.	n.a.	n.a.	4 CRC (2.9%)<br>(additional yield)	100%	92 (67%)	None
Copel [40]<br>2007	Cohort<br>Retrospective	No serious limitations	Good	Good	None	546	546	Single	Yes	No	n.a.	Yes<br>-41.80%	4- or<br>8-slice	100 mAs<br>200 mAs	Expert	Primary 2D	No	14	70%	7	33%	7 CRC<br>(additional yield)	91%	n.a.	None
Morrin [41]<br>1999	Cohort<br>Prospective	No serious limitations	Good	Good	None	40	40	Single	Yes	No	1.5	No	1- or<br>4-slice	120 mAs	n.a.	Primary 2D	No	0	n.a.	2	50%	0	n.a.	5 (13%)	None
Yucel [42]<br>2008	Cohort<br>Retrospective	No serious limitations	Good	Good	None	42	42	Single	No	Yes	1	No	64-slice	150 mAs<br>200 mAs	Expert	Primary 3D	No	12	n.a.	31	n.a.	1 CRC<br>(additional yield)	n.a.	26 (62%)	In 12 patients CTC was performed as first examination.<br>Out of 22 patients with polyps at CTC, only 5 underwent colonoscopy for verification.
Macari [43]<br>1999	Cohort<br>Prospective	No serious limitations	Good	Good	None	20	20	Single	No	No	2.5	No	1-slice	150 mAs	n.a.	Primary 2D	No	1	100%	1	100%	0	n.a.	n.a.	None
Neri [44]<br>2002	Cohort<br>Retrospective	No serious limitations	Good	Good	None	34	34	Single	Yes	No	2	Yes	1-slice	180 mAs	Expert	n.a.	No	11	100%	24	86%	13 CRC<br>(additional yield)	100%	11 hepatic lesions	None
Luo [45]<br>2002	Cohort<br>Prospective	No serious limitations	Good	Good	None	60	60	Single	Yes	No	n.a.	No	1-slice	100 mAs<br>120 mAs	n.a.	n.a.	No	2	n.a.	10	n.a.	1 CRC<br>(additional yield)	100%	n.a.	None
Lai [46]<br>2009	Cohort<br>Retrospective	No serious limitations	Good	Good	None	196	196	Single	Yes	No	n.a.	No	4-slice	100 mAs<br>200 mAs	Expert	n.a.	No	10	70%	27	29.60%	10 CRC<br>(additional yield)	100%	n.a.	None

CAD, computer-aided detection; CRC, colorectal cancer; CT, computed tomography; CTC, computed tomographic colonography; E-RADS, Extracolonic CT Colonography Reporting and Data System; GRADE, Grading of Recommendations Assessment Development and Evaluation; mAs, milliampere seconds; n.a., not available; PPV, positive predictive value.

Evidence table for clinical question 5 (CTC after positive faecal occult blood test):

Appendix e2 Computed tomographic colonography (CTC): evidence tables

Table e5 Computed tomographic colonography (CTC) performance in patients with positive fecal occult blood test (FOBT) or fecal immunochemical test (FIT).

First author	GRADE study characteristics					FOBT/FIT-positive screenees, n	Single- or multicenter	Catharsis	Tagging	Dose, mAs	Reconstruction interval, mm	Intravenous contrast	Reader experience	Reading strategy	CAD	Double reading	Reference standard	Cancers, n		Per-patient 6-mm+ lesions or cancer	
Year	Design	Quality	Consistency	Directness	Other modifying factors													CTC	By reference standard	CTC	By reference standard
Heresbach [105] 2011	Cohort	No serious limitations	Good	Good	None	50	Multicenter	Yes	Yes	50–75	1–1.25	No	Minimum 50 cases	Either	No	No	Segmental unblinded colonoscopy	–	–	–	–
Liedenbaum [106] 2009	Cohort	No serious limitations	Good	Good	None	302	Multicenter	No	Yes	<50	0.9–1	No	Minimum 100 cases	Primary 2D	No	Yes	Segmental unblinded colonoscopy	21	22	192	211
Liedenbaum [107] 2009	Cohort	No serious limitations	Good	Good	Same patients as reference [38]	302	Multicenter	No	Yes	<50	0.9–1	No	Minimum 100 cases	Primary 2D	No	Yes	Segmental unblinded colonoscopy	n.a.	n.a.	n.a.	n.a.
Regge [15] 2009	Cohort	No serious limitations	Good	Good	None	221	Multicenter	Yes	In '1/3	<50	0.6–1.5	No	Minimum 50 cases	Either	No	No	Segmental unblinded colonoscopy	–	–	96	111
Sali [108] 2010	Cohort	Single center and single radiologist	Good	Good	None	49	Single-center	Yes	No	50	1	No	Approximately 100 cases	Primary 2D	No	No	Segmental unblinded colonoscopy	2	2	21	22

CAD, computer-aided detection; FIT, fecal immunochemical test; FOBT, fecal occult blood test; n.a., not available.
